# Discrepancy between the *in vitro *and *in vivo *effects of murine mesenchymal stem cells on T-cell proliferation and collagen-induced arthritis

**DOI:** 10.1186/ar2939

**Published:** 2010-02-22

**Authors:** Evelien Schurgers, Hilde Kelchtermans, Tania Mitera, Lies Geboes, Patrick Matthys

**Affiliations:** 1 Laboratory of Immunobiology, Rega Institute, Faculty of Medicine, Katholieke Universiteit Leuven, Minderbroedersstraat 10, 3000 Leuven, Belgium

## Abstract

**Introduction:**

The goal of this study is to analyze the potential immunosuppressive properties of mesenchymal stem cells (MSC) on T cell proliferation and in collagen-induced arthritis (CIA). An additional aim is to investigate the role of interferon-γ (IFN-γ) in these processes.

**Methods:**

MSC were isolated from bone marrow of DBA/1 wild type and IFN-γ receptor knock-out (IFN-γR KO) mice and expanded *in vitro*. Proliferation of anti-CD3-stimulated CD4^+ ^T cells in the presence or absence of MSC was evaluated by thymidine incorporation. CIA was induced in DBA/1 mice and animals were treated with MSC by intravenous or intraperitoneal injections of wild type or IFN-γR KO MSC.

**Results:**

Purity of enriched MSC cultures was evaluated by flow cytometry and their ability to differentiate into osteoblasts and adipocytes. *In vitro*, wild type MSC dose-dependently suppressed anti-CD3-induced T cell proliferation whereas IFN-γR KO MSC had a significantly lower inhibitory potential. A role for inducible nitric oxide (iNOS), programmed death ligand-1 (PD-L1) and prostaglandin E2 (PGE_2_), but not indoleamine 2,3-dioxigenase (IDO), in the T cell inhibition was demonstrated. *In vivo*, neither wild type nor IFN-γR KO MSC were able to reduce the severity of CIA or the humoral or cellular immune response toward collagen type II.

**Conclusions:**

Whereas MSC inhibit anti-CD3-induced proliferation of T cells *in vitro*, an effect partially mediated by IFN-γ, MSC do not influence *in vivo *T cell proliferation nor the disease course of CIA. Thus there is a clear discrepancy between the *in vitro *and *in vivo *effects of MSC on T cell proliferation and CIA.

## Introduction

Bone marrow-derived mesenchymal stem cells (MSCs) are multipotent progenitor cells that can differentiate into cells of the mesenchymal lineage like bone, fat, and cartilage [1]. Due to these characteristics, they have been postulated as attractive candidates for cell-based tissue repair (for instance, to restore cartilage defects) [2,3]. MSCs have therefore been suggested as an innovative therapeutic tool for rheumatic diseases [4]. Besides their regenerative potential, MSCs have immunomodulatory properties by interaction with immunocompetent cells (reviewed in [5,6]). MSCs inhibit proliferation of T cells in response to mitogenic stimuli [7] and anti-CD3 and anti-CD28 antibody stimulation [8,9]. Multiple mechanisms have been proposed by which MSCs inhibit T-cell responses. Prostaglandin E_2 _(PGE_2_), nitric oxide (NO), indoleamine 2,3-dioxigenase (IDO), and programmed death ligand-1 (PD-L1) (also known as B7-H1) are among the most often postulated molecules to be involved in inhibition of T-cell proliferation by MSCs [10-12]. Besides the involvement of soluble factors, induction of T-cell anergy has emerged as an alternative mechanism of T-cell inhibition [13]. To suppress T-cell responses, MSCs first need to be activated by cytokines produced by activated T cells [14,15], like interferon-gamma (IFN-γ). Although IFN-γ has traditionally been considered a pro-inflammatory cytokine, evidence that IFN-γ can also fulfill potent immunomodulatory properties is accumulating [16]. Stimulation with IFN-γ can induce MSCs to inhibit T-cell proliferation [12,15]. *In vivo *data on MSC-mediated immunosuppression, however, are less conclusive. When graft-versus-host disease is induced in mice, treatment with MSCs does not always result in amelioration of the disease [17-19]. T cell-mediated autoimmune diseases like experimental autoimmune encephalomyelitis and experimental autoimmune enteropathy demonstrated an amelioration of symptoms after treatment with MSCs [20-22]. Treatment of collagen-induced arthritis (CIA), an animal model for rheumatoid arthritis, with MSCs has also been investigated. While three studies report amelioration of arthritic symptoms [23-25], others were unable to see beneficial effects of MSC treatment on the development of CIA [26,27]. In patients with rheumatoid arthritis, MSCs were able to suppress collagen-specific T-cell responses *in vitro *[28]. To strengthen the experimental background for future therapy with MSCs, we addressed the effect of MSCs on *in vitro *and *in vivo *T-cell proliferation and on CIA in this study. In addition, we investigated the role of IFN-γ by using MSCs isolated from IFN-γ receptor knockout (IFN-γR KO) mice.

## Materials and methods

### Isolation and expansion of mesenchymal stem cells

DBA/1 mice were bred in the Animal Centre of the University of Leuven. Bone marrow from 4- to 6-week-old DBA/1 and DBA/1 IFN-γR KO mice was flushed out of the femurs and tibias by using phosphate-buffered saline (PBS) supplemented with 2% fetal calf serum (FCS) (Gibco, now part of Invitrogen Corporation, Carlsbad, CA, USA). Cells were washed once with PBS 2% FCS and plated at a concentration of 0.6 to 0.8 × 10^6 ^cells/cm^2 ^in Murine Mesencult medium (StemCell Technologies, Vancouver, BC, Canada) supplemented with 100 U/ml penicillin (Continental Pharma, Brussels, Belgium) and 100 μg/ml streptomycin (Continental Pharma). Cells were cultured in a humidified atmosphere with 5% CO_2 _at 37°C. Half of the medium was replaced after 2 days and thereafter twice a week for 3 weeks. When the colonies that had formed reached confluence, adherent cells were collected following a 5-minute incubation at 37°C with 0.05% trypsin/ethylenediaminetetraacetic acid (EDTA) (Gibco) and replated. MSCs of C57BL/6 origin were kindly provided by Darwin J Prockop and Catherine Verfaillie.

### Flow cytometric characterization and differentiation of mesenchymal stem cells

MSCs were harvested by incubation with trypsin/EDTA and counted. MSCs were washed with PBS 2% FCS, stained with the indicated antibodies for 30 minutes and washed twice with PBS 2% FCS. For the biotin-conjugated antibody, a second staining step with streptavidin conjugated to fluorescein isothiocyanate (FITC) was performed. Finally, the cells were fixed with 0.37% formaldehyde in PBS. The following antibodies were purchased from eBioscience (San Diego, CA, USA): Sca-1-FITC (stem cell antigen-1 [Ly-6A/E]), CD34-FITC, MHC-I-FITC, CD31-phycoerythrin (platelet endothelial cell adhesion molecule [PCAM]-PE), CD73-PE (ecto-5'-nuleotidase), MHC-II-PE, CD11b-PE, CD105-biotin (endoglin), and CD45-phycoerythrin-cyanine-5 (PE-Cy5). Flow cytometric analysis was performed on a FACSCalibur flow cytometer with CellQuest^® ^software (BD Biosciences, San Jose, CA, USA). For differentiation, MSCs were plated in six-well plates and grown to confluence. Osteogenesis and adipogenesis were induced as described previously [29] and [30], respectively).

### Anti-CD3-induced cell proliferation

CD4^+ ^T cells and accessory cells (ACs) were isolated from DBA/1 mice and cultured in RPMI medium as described previously [31]. CD4^+ ^T cells (5 × 10^4 ^per well) were cultured in flat-bottomed 96-well plates with mitomycin-c-treated (Sigma-Aldrich, St. Louis, MO, USA) ACs (5 × 10^4 ^per well) and 3 μg/ml anti-CD3 antibody in the presence or absence of the indicated numbers of mitomycin-c-treated MSCs. The cultures were incubated for 72 hours at 37°C in 5% CO_2 _and pulsed for the last 16 hours with 1 μCi of [^3^H]TdR and harvested. The suppressive capacity of the MSCs is represented by the percentage inhibition. Alternatively, CD4^+ ^T cells were labeled with carboxyfluorescein succinimidyl ester (CFSE) (Invitrogen Corporation, Carlsbad, CA, USA) before culture to analyze cell proliferation. T cells were resuspended in PBS 5% FCS at a concentration of 1 to 2 × 10^6 ^cells/ml and incubated with CFSE (final concentration of 50 μM) for 5 minutes at room temperature. Cells were washed three times with PBS 5% FCS and resuspended in culture medium at the indicated concentrations. For restoration of T-cell proliferation, co-cultures were grown in the presence of 200 μM 1-methyl-DL-tryptophan (Sigma-Aldrich), 10 μM indomethacin (Sigma-Aldrich), or 10 μM GW274150 (Alexis Biochemicals, Farmingdale, NY, USA).

### Measurement of *in vivo *T-cell proliferation

*In vivo *T-cell proliferation was measured using the Click-iT™ EdU Flow Cytometry Assay Kit (Invitrogen Corporation). EdU (5-ethynyl-2'-deoxyuridine) is a nucleoside analog to thymidine and is incorporated into DNA during active DNA synthesis. One milligram of EdU in 100 μl of PBS was injected intraperitoneally into each mouse. After 4 hours, mice were sacrificed and lymph nodes (axillary, inguinal, and mesenteric) and spleens were harvested. Single-cell suspensions were obtained as described above and were incubated for 15 minutes with the Fc receptor-blocking antibodies anti-CD16 and anti-CD32 (CD16/CD32; Miltenyi Biotec, Bergisch Gladbach, Germany). Cells were washed with PBS 1% bovine serum albumin (BSA) and incubated with anti-CD4-FITC and anti-CD8-Per-CP antibodies (eBioscience) for 30 minutes and then washed twice with PBS 1% BSA followed by detection of incorporated EdU in accordance with the manufacturer's instructions. Flow cytometric analysis was performed on a FACSCalibur flow cytometer with CellQuest^® ^software.

### Quantitative polymerase chain reaction

RNA extraction, cDNA synthesis, and real-time quantitative polymerase chain reaction (PCR) for inducible nitric oxide (iNOS), IDO, cyclo-oxigenase-2 (COX-2), and PD-L1 (assay ID Mm00440485_m1, Mm00492586_m1, Mm01307334_g1, and Mm00452054_m1, respectively; Applied Biosystems, Foster City, CA, USA) were performed as described previously [32].

### Bio-Plex protein array system

Expression of cytokines (that is, interleukin-2 [IL-2], IL-5, IL-6, IL-10, IL-17, and IFN-γ) was determined by the Bio-Plex 200 system, Bio-Plex mouse Cytokine 8-plex assay, Bio-Plex mouse IL-6 assay, and Bio-Plex mouse IL-17 assay (Bio-Rad Laboratories, Inc., Hercules, CA, USA).

### Collagen-induced arthritis induction and treatment protocols

Experiments were performed in 8- to 12-week-old DBA/1 mice. CIA was induced and clinically assessed as described previously [33]. To test the effect of MSCs on the disease course of CIA, mice were injected intravenously or intraperitoneally with 1 × 10^6 ^MSCs in 100 μl of sterile PBS at the indicated time points. Controls received injections of an equal volume of PBS at the same time points. All animal experiments were approved by the local ethics committee (University of Leuven).

### Measurement of anti-CII antibodies and delayed-type hypersensitivity to CII

Blood samples were taken from the orbital sinus or by heart puncture and were allowed to clot at room temperature for 1 hour and at 4°C overnight. Individual sera were tested for antibodies directed to chicken collagen type II (CII) by enzyme-linked immunosorbent assay as described earlier [31]. For evaluation of delayed-type hypersensitivity (DTH) reactivity, CII/complete Freund's adjuvant (CFA)-immunized mice were subcutaneously injected with 20 μg of CII/20 μl of PBS in the right ear and with 20 μl of PBS in the left ear. DTH response was calculated as the percentage swelling (the difference between the increase of thickness of the right ear and the left ear, divided by the thickness of the left ear, multiplied by 100).

### Statistical analysis

Data are expressed as the mean (standard error of the mean). Differences were analyzed by the Mann-Whitney *U *test. A *P *value of not more than 0.05 was considered significant.

## Results

### Generation of mesenchymal stem cells and phenotypical analysis

**Figure 1:**
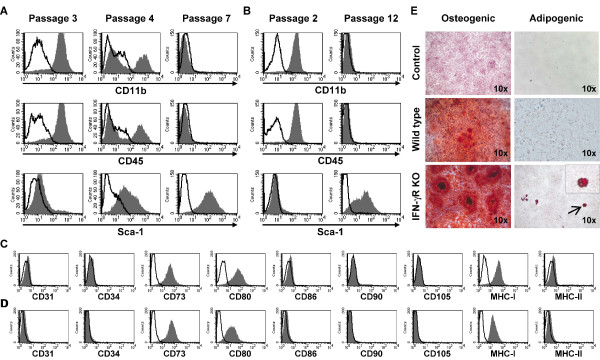
**Phenotype and differentiation potential of mesenchymal stem cells (MSCs)**. Bone marrow cells of DBA/1 wild-type and interferon-gamma receptor knockout (IFN-γR KO) mice were cultured in Murine Mesencult medium and phenotyped. **(a-d) **MSCs were incubated with the indicated antibodies and analyzed by flow cytometry. Grey histograms show stained cells, and black lines represent cells incubated with isotype controls. Wild-type MSCs were analyzed at passages 3, 4, and 7 (a) and IFN-γR KO MSCs were analyzed at passages 2 and 12 (b) for expression of CD11b, CD45, and Sca-1. Likewise, other phenotypic markers were analyzed on wild-type (c) and IFN-γR KO (d) MSCs. **(e) **MSCs were cultured to confluency in Murine Mesencult medium and then transferred to adipogenic or osteogenic differentiation medium for 21 days, followed by Oil Red O or Alizarin Red staining, respectively (original magnification × 10). The inset in the lower right panel represents an enlargement of the adipocyte indicated by the arrow.

MSCs were generated from bone marrow cells of DBA/1 wild-type and DBA/1 IFN-γR KO mice. After removal of nonadherent cells, colonies were formed. These colonies were morphologically heterogeneous until passage 5 or 6, consisting of both round and fibroblast-like cells. Heterogeneity was also evident from phenotypical analysis of the cell cultures by flow cytometry. During the first 2 to 4 passages, cell cultures consisted predominantly of CD11b^+ ^and CD45^+ ^hematopoietic cells (Figure 1a, b). The original population of bone marrow cells was enriched with MSCs during subsequent passages. From passage 7 onward, a homogenous CD11b^-^CD45^-^Sca-1^+ ^population of MSCs was reached for both wild-type and IFN-γR KO cultures (passages 7 and 12 are depicted in Figure 1a, b). Additional flow cytometric analysis demonstrated that the MSC cultures from passage 7 were positive for CD73, CD80, and MHC-I and negative for CD31, CD34, CD86, CD90, CD105, and MHC-II (WT MSCs, Figure 1c; IFN-γR KO MSCs, Figure 1d).

### The isolated mesenchymal stem cells differentiate into osteogenic and adipogenic lineages

To assess the multipotentiality of the cultured mouse MSCs, cells were subjected to *in vitro *osteogenic and adipogenic differentiation assays. In osteogenic medium, the MSCs of both wild-type and IFN-γR KO origin formed aggregates and showed enhanced calcium deposition as revealed by Alizarin Red stain (Figure 1e, middle and lower left panels) as compared with control cultures grown in medium without additives. By culturing the MSCs in adipogenic medium, only MSCs from DBA/1 IFN-γR KO mice showed some adipogenic differentiation (Figure 1e, lower right panel), whereas MSCs of DBA/1 wild-type origin showed no adipogenic differentiation (Figure 1c, middle right panel).

### Mesenchymal stem cells suppress anti-CD3-induced T-cell proliferation *in vitro *by a mechanism involving interferon-gamma, inducible nitric oxide, and cyclo-oxigenase-2

**Figure 2:**
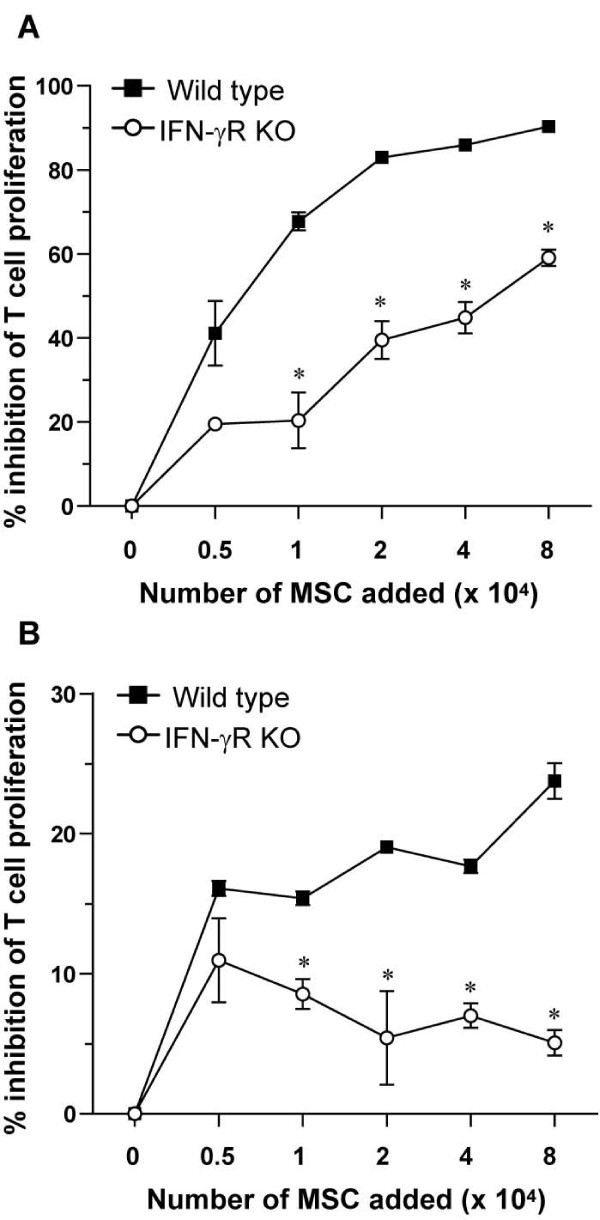
**Mesenchymal stem cells (MSCs) inhibit the anti-CD3-induced proliferation of CD4^+ ^T cells *in vitro***. **(a) **CD4^+ ^T cells (5 × 10^4 ^cells) and accessory cells (5 × 10^4 ^cells) were incubated with 3 μg/ml anti-CD3 antibody and the indicated numbers of mitomycin c-treated wild-type or interferon-gamma receptor knockout (IFN-γR KO) MSCs for 72 hours and pulsed for the last 16 hours with 1 μCi of [^3^H]TdR. The percentage inhibition (100 × [(radioactivity in cultures without MSCs -- radioactivity in cultures with MSCs)/radioactivity in cultures without MSCs]) by increasing numbers of MSCs is shown. Each result represents the mean of four cultures ± standard error of the mean (SEM). Results are representative of two independent experiments. * *P *< 0.05 for comparison with wild-type MSCs (Mann-Whitney *U *test). **(b) **Carboxyfluorescein succinimidyl ester (CFSE)-labeled CD4^+ ^T cells (5 × 10^4 ^cells) and accessory cells (5 × 10^4 ^cells) were incubated with 3 μg/ml anti-CD3 antibody and the indicated numbers of mitomycin c-treated wild-type or IFN-γR KO MSCs for 72 hours. The proliferation of CD4^+ ^T cells was analyzed by detection of CFSE dilution by flow cytometry. The percentage inhibition (100 × [(percentage of proliferating CD4^+ ^cells not treated with MSCs -- percentage of proliferating CD4^+ ^cells treated with MSCs)/percentage of proliferating CD4^+ ^cells not treated with MSCs]) by increasing numbers of MSCs is shown. Each result represents the mean of three cultures ± SEM. Results are representative of two independent experiments. * *P *< 0.05 for comparison with wild-type MSCs (Mann-Whitney *U *test).

To investigate the immunosuppressive potential of MSCs *in vitro*, we tested their effect on the anti-CD3-induced proliferation of CD4^+ ^T cells. T cells were stimulated *in vitro *with anti-CD3 antibody in the absence or presence of MSCs and their proliferation was analyzed by thymidine incorporation. MSCs of wild-type origin dose-dependently inhibited anti-CD3-induced T-cell proliferation (Figure 2a). IFN-γR KO MSCs had a significantly lower inhibitory capacity (Figure 2a). Proliferation was also measured by analysis of CFSE-labeled CD4^+ ^T cells. Similarly, a lower suppressive capacity of IFN-γR KO MSCs as compared with wild-type MSCs was seen (Figure 2b).

**Figure 3:**
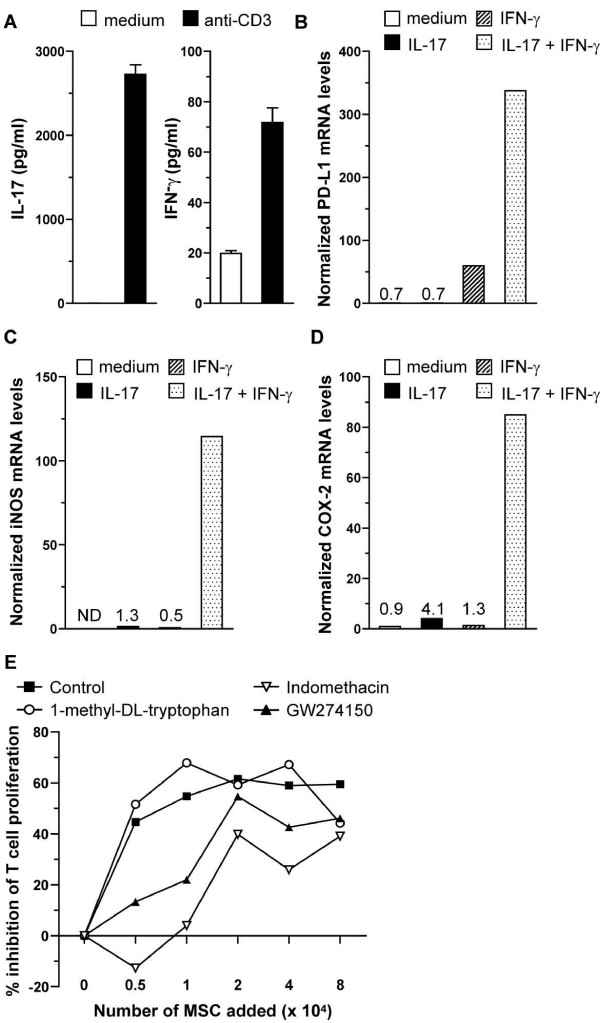
**Mesenchymal stem cells (MSCs) inhibit the proliferation of CD4^+ ^T cells *in vitro *by induction of nitric oxide and prostaglandin E_2 _(PGE_2_)**. **(a) **CD4^+ ^T cells, in the presence of accessory cells, were stimulated with 3 μg/ml anti-CD3 for 48 hours. Interleukin-17 (IL-17) and interferon-gamma (IFN-γ) levels in the supernatant of these cultures were analyzed by Bio-Plex protein array system. Bars represent averages of three values ± standard error of the mean. **(b-d) **Wild-type MSCs were stimulated with IL-17 (20 ng/mL) or IFN-γ (100 U/mL) or both for 48 hours. cDNA samples were prepared and subjected to quantitative polymerase chain reaction analysis. The relative quantity of target mRNA levels was normalized for 18S RNA. Relative levels of programmed death ligand-1 (PD-L1) (b), inducible nitric oxide (iNOS) (c), and cyclo-oxigenase-2 (COX-2) (d) are shown. Bars represent averages of two values. ND, not detectable. **(e) **CD4^+ ^T cells (5 × 10^4 ^cells) and accessory cells (5 × 10^4 ^cells) were incubated with 3 μg/ml anti-CD3 antibody and the indicated number of mitomycin c-treated wild-type MSCs for 72 hours and pulsed for the last 16 hours with 1 μCi of [^3^H]TdR. Co-cultures were grown in the absence (control) or presence of 200 μM 1-methyl-DL-tryptophan, 10 μM indomethacin, or 10 μM GW274150. The percentage inhibition (100 × [(radioactivity in cultures without MSCs -- radioactivity in cultures with MSCs)/radioactivity in cultures without MSCs]) by increasing numbers of MSCs is shown.

These data demonstrate the importance of IFN-γ signaling in MSCs to suppress T-cell proliferation. To investigate which molecules are involved in the suppression of proliferation, quantitative PCR was performed on IL-17- and IFN-γ-stimulated wild-type MSCs. These stimuli were chosen based on their upregulated expression in CD4^+ ^T cells by stimulation with anti-CD3 antibodies (Figure 3a) and because these cytokines have been shown to synergistically induce the expression of iNOS [34] and IDO [35] in fibroblasts. The expression of iNOS, IDO, PD-L1, and COX-2, molecules involved in inhibition of T-cell proliferation and known to be induced by IFN-γ in MSCs [11], was analyzed. Unstimulated MSCs expressed no or low levels of these inhibitory factors (Figure 3b-d). Upon stimulation with IL-17 or IFN-γ alone, expression of PD-L1 (Figure 3b), iNOS (Figure 3c), and COX-2 (Figure 3d) was upregulated mildly. However, when IL-17 and IFN-γ were added simultaneously, expression levels of PD-L1, iNOS, and COX-2 (Figure 3b-d) were synergistically upregulated. IDO mRNA could not be detected in unstimulated or stimulated MSCs (data not shown). These data indicate that IFN-γ acts synergistically with IL-17 to upregulate expression of PD-L1, iNOS, and COX-2 in MSCs, making these molecules candidate mediators of T-cell inhibition. The involvement of iNOS and COX-2 in inhibition of T-cell proliferation was demonstrated by the addition of inhibitors of these enzymes - GW274150 and indomethacin [8,36], respectively - to the co-cultures. The addition of these inhibitors resulted in the abrogation of the inhibition of T-cell proliferation by wild-type MSCs (Figure 3e). The addition of the IDO inhibitor 1-methyl-DL-tryptophan (1-MT) did not affect the inhibition conferred by MSCs (Figure 3e).

### Mesenchymal stem cell treatment has no effect on the development of collagen-induced arthritis

**Figure 4:**
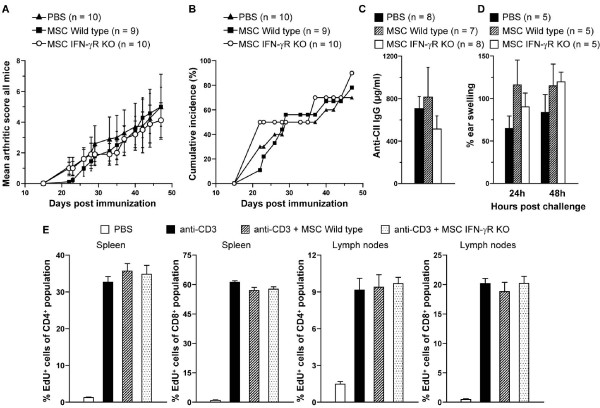
**Treatment with mesenchymal stem cells (MSCs) of wild-type or interferon-gamma receptor knockout (IFN-γR KO) origin does not influence the development of collagen-induced arthritis in DBA/1 mice**. Mice were immunized on day 0 with collagen type II (CII) in complete Freund's adjuvant and injected intravenously with MSCs on day 16 and day 23. The mean arthritic score **(a) **and the cumulative incidence of arthritis **(b) **in DBA/1 mice treated with phosphate-buffered saline (PBS), wild-type MSCs, or IFN-γR KO MSCs are shown. Error bars represent standard error of the mean (SEM). **(c) **On day 46, sera of individual mice were analyzed for total anti-CII IgG. Histograms represent averages ± SEM. **(d) **Forty-two days after immunization, five mice in each group were challenged with 10 μg of CII in the right ear and vehicle in the left ear. Delayed-type hypersensitivity responses were measured as the percentage of swelling (100 × [(thickness of the right ear -- thickness of the left ear)/thickness of the left ear]) at the indicated times. Histograms indicate averages ± SEM. **(e) **On day 19 after immunization with CII in complete Freund's adjuvant, DBA/1 wild-type mice were injected intravenously with 1 × 10^6 ^wild-type MSCs, IFN-γR KO MSCs, or PBS, followed by an administration of 10 μg of anti-CD3 antibody on day 20. On day 21, *in vivo *T-cell proliferation was measured by detection of 5-ethynyl-2' -deoxyuridine (EdU) in the T-cell populations in the spleen and lymph nodes by fluorescence-activated cell sorting analysis. The percentages of EdU-positive cells in the CD4^+ ^and CD8^+ ^populations in the spleen and lymph nodes are shown. Histograms represent averages of four mice ± SEM.

To test the possible involvement of MSCs in CIA, DBA/1 mice were immunized with CII in CFA on day 0 and injected intravenously with wild-type or IFN-γR KO MSCs at different time points (Table 1). In a first experiment, day -1 was chosen for treatment with MSCs because experiments previously performed in our laboratory demonstrated that one single injection of CD4^+^CD25^+ ^regulatory T (T_reg_) cells at day -1 significantly inhibited CIA [37]. In fact, in this experiment, a group of mice that received T_reg _cells were included. Injection of either wild-type or IFN-γR KO MSCs at day -1 did not affect the severity or incidence of arthritis, whereas injection of T_reg _cells did reduce the severity of CIA. In two subsequent experiments, we considered treating the mice at later time points, when inflammation was already ongoing. Thus, MSCs were administered at day 16 (experiment 2 in Table 1) or at day 16 and 23 post-immunization (experiment 3 in Table 1 and Figure 4). Treatment of the mice did not influence the disease severity or the incidence of arthritis development (Table 1 and Figure 4a, b) as compared with PBS-treated control animals. To verify whether the failure of MSCs to affect clinical scores of arthritis was also reflected in cellular and humoral autoimmune responses, DTH and total anti-CII IgG were analyzed. Anti-CII IgG titers were similar between MSC-treated and PBS-treated mice (Figure 4c). In addition, DTH responses, as evident from the percentage of swelling upon challenge with CII, were not different between MSC-treated and control animals (Figure 4d). T-cell proliferation was also measured in these mice by injection of 10 μg of anti-CD3 antibody. The results revealed no differences in CD4^+ ^and CD8^+ ^T-cell proliferation in spleens and lymph nodes when arthritic mice were injected intravenously with wild-type or IFN-γR KO MSCs (Figure 4e). However, since T-cell activation is a combination of proliferation and cytokine production, the sera of anti-CD3-injected and MSC-treated mice were analyzed for cytokines. The serum of mice was pooled per group and analyzed for the T-cell cytokines IL-2, IL-5, IL-6, IL-10, and IFN-γ. The injection of anti-CD3 antibody caused a profound increase in cytokine levels in the sera of these mice. Treatment with wild-type or IFN-γR KO MSCs, however, did not result in a decrease of IL-2, IL-5, and IL-10 but slightly decreased the levels of IL-6 and IFN-γ (data not shown).

**Table 1:** Cumulative incidence and mean scores of arthritis in mice treated with wild-type or IFN-γR KO DBA/1 MSCs or with C57BL/6 MSCs^a^

Experiment	Route^b^	Administration time^c^	Treatment^d^	Cumulative incidence^e^, fraction (percentage)	Score of arthritis, mean ± SEM^e, f^
1	i.v.	Day -1	Control	4/8 (50%)	2.0 ± 0.9
			Wild-type MSCs	1/2 (50%)	1.5 ± 1.5
			IFN-γR KO MSCs	3/7 (43%)	1.6 ± 1.0
			T_reg _cells	2/8 (25%)	0.4 ± 0.3
2	i.v.	Day 16	Control	4/10 (40%)	1.3 ± 0.7
			Wild-type MSCs	6/8 (75%)	2.1 ± 0.6
			IFN-γR KO MSCs	4/10 (40%)	0.8 ± 0.4
3	i.v.	Days 16 and 23	Control	5/10 (50%)	3.0 ± 1.4
			Wild-type MSCs	5/9 (56%)	2.5 ± 0.9
			IFN-γR KO MSCs	5/10 (50%)	2.0 ± 0.8
4	i.p.	Days 16, 23, and 30	Control	7/8 (88%)	5.1 ± 1.5
			Wild-type MSCs	6/7 (86%)	5.3 ± 1.2
			IFN-γR KO MSCs	8/9 (89%)	4.6 ± 1.6
5	i.p.	Days 16, 23, and 30	Control	7/11 (64%)	1.3 ± 0.5
			C57BL/6 MSCs	8/11 (73%)	2.7 ± 1.2

^a^Mice were immunized with collagen type II in complete Freund's adjuvant on day 0 and were treated^b ^either intravenously (i.v.) or intraperitoneally (i.p.) on the indicated time points^c ^with wild-type mesenchymal stem cells (MSCs), interferon-gamma receptor knockout (IFN-γR KO) MSCs, C57BL/6 MSCs, regulatory T (T_reg_) cells, or phosphate-buffered saline (control group)^d^. ^e^Arthritic incidence and score of arthritis in all groups at day 35. ^f^Arthritic scores were not significantly different between control treatment and treatment with wild-type or IFN-γR KO DBA/1 MSCs or C57BL/6 MSCs or with T_reg _cells. SEM, standard error of the mean.

Since in recently reported studies MSCs that successfully affected CIA were injected intraperitoneally [23,24], we performed an additional experiment in which wild-type or IFN-γR KO MSCs were administered intraperitoneally. Similarly to the intravenous administration, intraperitoneal treatment with MSCs did not influence the disease severity or incidence of arthritis compared with PBS-treated control mice (experiment 4 in Table 1). Here again, anti-CII IgG antibody levels were not different compared with controls (data not shown). The MSCs used in reference [23] were, however, of C57BL/6 origin. To exclude the possibility that the difference in treatment outcome depends on the mouse strain from which the MSCs are isolated, we performed an additional experiment in which MSCs of C57BL/6 origin were intraperitoneally injected. Again, there was no difference in cumulative incidence and mean arthritic score between the C57BL/6 MSC-treated and control-treated mice (experiment 5 in Table 1). The results of all experiments are summarized in Table 1.

## Discussion

Besides their inherent ability to differentiate into mesenchymal cell lineages [1] and their potential to repair damaged tissue [2,3], MSCs have been shown to exert immunosuppressive properties on T cells. For this reason, studies to test the use of MSCs for treatment of several T cell-mediated inflammatory diseases have been conducted. In CIA, the effect of MSCs on the disease severity was not clear-cut [23-27]. Therefore, in the present study, we assessed the effect of MSCs on *in vitro *and *in vivo *T-cell proliferation as well as on CIA. By using MSCs of both IFN-γR KO and wild-type origin, we also addressed the role of IFN-γ in the immunomodulatory properties of MSCs.

The obtained MSCs demonstrated a phenotype that matches the generally accepted phenotype for murine MSCs, being positive for CD73 and Sca-1 and negative for CD11b, CD31, CD34, CD45, and CD90 [38]. Differentiation toward osteocytes could be demonstrated in wild-type and IFN-γR KO MSCs and was equally potent in the two cell types. The differentiation of MSCs toward adipocytes was much less pronounced. A possible explanation for this observation can be the DBA/1 origin of the MSCs. Indeed, it has been demonstrated that DBA/1 MSCs formed osteocytes very potently but differentiation into adipocytes was much more difficult in this mouse strain compared with other strains [30].

Co-culturing anti-CD3-stimulated T cells and MSCs clearly resulted in an inhibition of T-cell proliferation in an IFN-γ-dependent way. This observation is in agreement with other reports emphasizing the role of IFN-γ in MSC-mediated immunosuppression [11,12,15,39]. When T-cell proliferation was analyzed by radioactive thymidine incorporation, a higher suppression of T-cell proliferation could be achieved compared with analysis by CFSE dilution. A possible explanation for this difference might be the time frame during which proliferation was analyzed since MSCs need IFN-γ from activated T cells to suppress immune responses. CFSE is present from the start of the culture, whereas [^3^H]TdR is added only during the last 16 hours of cell culture. Thus, the CFSE-based method measures all T-cell proliferation, whereas the [^3^H]TdR method measures only late T-cell proliferation, when the inhibition by MSCs is ongoing. Irrespective of the method that is used for measurement of T-cell proliferation, IFN-γR KO MSCs display a defect in their potential to inhibit T-cell proliferation.

We identified a possible role for NO, PD-L1, and PGE_2 _but not IDO in the inhibition of T-cell proliferation by MSCs. Several independent reports identified NO as being one of the major mediators of T-cell suppression by MSCs [10,15,39,40], whereas controversy about the involvement of IDO still exists [8,11,41,42]. The same holds true for PD-L1 and PGE_2_, with reports supporting [11,12] and refuting [8,11] a role for these two T-cell inhibitors in T-cell proliferation inhibition. In addition, we could demonstrate that IL-17, in synergy with IFN-γ, can induce the expression of iNOS, COX-2, and PD-L1 in MSCs. Thus, IL-17 and IFN-γ from activated T cells can induce MSCs to suppress ongoing T-cell responses *in vitro*.

Treatment with MSCs did not affect the disease course of CIA. The pathogenesis of CIA, an animal model of human rheumatoid arthritis, depends on both CII-specific T cells and antibody responses against CII [43,44]. Both of these specific immune responses against CII remained unaffected upon transfer of MSCs. In five experiments, MSCs administered intravenously or intraperitoneally did not affect the development of arthritis (Table 1). A possible explanation for the discrepancy between the *in vitro *and *in vivo *settings might be that the intravenously injected MSCs do not reach the spleen and lymph nodes and are therefore unable to inhibit the T-cell proliferation and CIA. In rats, radioactively labeled MSCs distribute mainly to the lungs and liver when intravenously administered [45,46]. Only small amounts of radioactivity could be detected in the spleen. Moreover, evidence exists that MSCs lose their homing ability to bone marrow after 48 hours of culture [47]. Since the MSCs used in this report were cultured for several weeks, cells may have lost their ability for homing to lymphoid organs. This homing ability can be improved by genetic manipulation of MSCs before transfer, as evident from a recent study reporting improved homing of MSCs to bone marrow in mice after overexpression of the chemokine receptor CXCR4 [48].

These results are in contrast to three reports [23-25] demonstrating that the administration of MSCs has a beneficial effect on disease severity in CIA. Other reports, however, support our data. Choi and colleagues [27] have shown that MSCs administered intravenously do not suppress the development of arthritis, unless they were transduced with IL-10, indicating that MSCs as such are not immunosuppressive in CIA. Similarly, in another study, it is reported that intravenous administration of the immortalized MSC cell line C3H10T1/2 to immunized mice had no effect on the development of CIA [26]. The treatment protocols and results of these studies are summarized in Table 2. Thus, overall, the results obtained with MSC treatment for CIA are inconclusive. This is in contrast to transfer of T_reg _cells for the treatment of CIA. When mice are injected with 1 × 10^6 ^T_reg _cells either before immunization or after disease onset, the severity of arthritis is dramatically diminished (Table 1 and [37,49]).

**Table 2:** Chronologic overview of literature describing the effect of mesenchymal stem cells on collagen-induced arthritis

	MSC source				MSC administration			
			
Reference	Organism^a^	Strain^b^	Organ^c^	Transfection^d^	Route^e^	Dose^f^	Time^g^	Result^h^
[26]	Mouse	C3	Cell line	n.a.	i.v.	1 × 10^6^	0	0
							21	0
						4 × 10^6^	0	0
							21	-
				IL-10	i.v.	1 × 10^6^	0	0
[23]	Mouse	C57Bl/10	BM	n.a.	i.p.	5 × 10^6^	0	+
							21	+
[27]	Mouse	DBA/1	BM	n.a.	i.v.	1 × 10^6^	21+28+35	0
				IL-10	i.v.	1 × 10^6^	21+28+35	+
[24]	Human	n.a.	Adipose	n.a.	i.p.	1 × 10^6^	5 a.d.o.	+
	Mouse	C57Bl/6	Adipose	n.a.	i.p.	1 × 10^6^	5 a.d.o.	+
	Mouse	DBA/1	Adipose	n.a.	i.p.	1 × 10^6^	5 a.d.o.	+
[25]	Rat	n.s.	BM	n.a.	i.v.	2 × 10^6^	1 a.d.o.	+

Mesenchymal stem cells (MSCs) in the studies described were isolated from different organisms^a^, strains^b^, and organs^c ^and transfected (interleukin-10 (IL-10)) or not (n.a.) with IL-10 expression vectors^d^. MSCs were administered intravenously (i.v.) or intraperitoneally (i.p.)^e ^at specified doses^f ^and administered at different time points (that is, days after immunization^g^). ^h^Results of the studies were summarized as follows: 0, no effect; -, worsening of symptoms; +, beneficial effect on arthritis symptoms. a.d.o., after disease onset; BM, bone marrow; n.a., not applicable; n.s., not specified.

## Conclusions

Our data demonstrate that murine bone marrow-derived MSCs potently inhibit *in vitro *T-cell proliferation in an IFN-γ-dependent mechanism that involves NO and PGE_2_. These *in vitro *data, however, could not be extrapolated to an *in vivo *situation. Neither *in vivo *anti-CD3-induced T-cell proliferation nor the development of CIA was affected by MSC treatment. Thus, although MSCs provide promising tools for the treatment of several autoimmune diseases, prudence is called for in extrapolating *in vitro *and animal data to the human situation.

## Abbreviations

AC: accessory cell; BSA: bovine serum albumin; CFA: complete Freund's adjuvant; CFSE: carboxyfluorescein succinimidyl ester; CIA: collagen-induced arthritis; CII: collagen type II; COX-2: cyclo-oxigenase-2; DTH: delayed-type hypersensitivity; EDTA: ethylenediaminetetraacetic acid; EdU: 5-ethynyl-2'-deoxyuridine; FCS: fetal calf serum; FITC: fluorescein isothiocyanate; IDO: indoleamine 2,3-dioxigenase; IFN-γ: interferon-gamma; IFN-γR KO: interferon-gamma receptor knockout; IL: interleukin; iNOS: inducible nitric oxide; MSC: mesenchymal stem cell; NO: nitric oxide; PBS: phosphate-buffered saline; PCR: polymerase chain reaction; PD-L1: programmed death ligand-1; PE: phycoerythrin; PGE_2_: prostaglandin E_2_; T_reg_: regulatory T.

## Competing interests

The authors declare that they have no competing interests.

## Authors' contributions

ES contributed to isolation and characterization of MSCs; MSC stimulation, quantitative PCR, and Bio-Plex; CIA induction and evaluation; humoral and cellular responses; analysis of T-cell proliferation; design of the study; and manuscript preparation. TM and HK contributed to MSC stimulation, quantitative PCR, and Bio-Plex; CIA induction and evaluation; humoral and cellular responses; and analysis of T-cell proliferation. LG contributed to CIA induction and evaluation and to analysis of T-cell proliferation. PM contributed to the design of the study and to manuscript preparation. All authors contributed to interpretation of the data. All authors read and approved the final manuscript.
